# Machine learning-aided risk stratification in Philadelphia chromosome-positive acute lymphoblastic leukemia

**DOI:** 10.1186/s40364-021-00268-x

**Published:** 2021-02-18

**Authors:** Satoshi Nishiwaki, Isamu Sugiura, Daisuke Koyama, Yukiyasu Ozawa, Masahide Osaki, Yuichi Ishikawa, Hitoshi Kiyoi

**Affiliations:** 1 Department of Advanced Medicine, Nagoya University Hospital, 65 Tsurumai-cho Showa-ku, Nagoya, 466-8560 Japan; 2 Division of Hematology and Oncology, Toyohashi Municipal Hospital, Toyohashi, Japan; 3 Department of Hematology, Japanese Red Cross Nagoya Daiichi Hospital, Nagoya, Japan; 4 Department of Hematology and Oncology, Nagoya University Graduate School of Medicine, Nagoya, Japan

**Keywords:** eXtreme gradient boosting algorithm, Machine learning, Philadelphia chromosome-positive acute lymphoblastic leukemia, Prognostic factor, Survival stratification

## Abstract

We used the eXtreme Gradient Boosting algorithm, an optimized gradient boosting machine learning library, and established a model to predict events in Philadelphia chromosome-positive acute lymphoblastic leukemia using a machine learning-aided method. A model was constructed using a training set (80%) and prediction was tested using a test set (20%). According to the feature importance score, *BCR-ABL* lineage, polymerase chain reaction value, age, and white blood cell count were identified as important features. These features were also confirmed by the permutation feature importance for the prediction using the test set. Both event-free survival and overall survival were clearly stratified according to risk groups categorized using these features: 80 and 100% in low risk (two or less factors), 42 and 47% in intermediate risk (three factors), and 0 and 10% in high risk (four factors) at 4 years. Machine learning-aided analysis was able to identify clinically useful prognostic factors using data from a relatively small number of patients.

**Supplementary Information:**

The online version contains supplementary material available at 10.1186/s40364-021-00268-x.

**To the Editor**

Several prognostic factors for Philadelphia chromosome-positive acute lymphoblastic leukemia (Ph + ALL) have been identified, such as minimal residual disease (MRD) [1, 2], chromosomal abnormalities [3], and genetic lesions [4]. However, further exploration is needed to identify the high-risk group in Ph + ALL The eXtreme Gradient Boosting (XGBoost) algorithm draws attention as an interpretable machine learning model [5], and is considered to be useful for identifying new prognostic factors for Ph + ALL.

## XGBoost model

Using a dataset of 59 adult Ph + ALL patients [6], we attempted to identify further risk factors using the XGBoost model [7] (TableS1 and S2). When the trained model was applied to the test set, the mean accuracy was 0.67, and the macro-average precision, recall, and f1-scores were 0.71, 0.78, and 0.66, respectively. The cross-validated accuracy was 0.66 (standard deviation 0.072). The area under the receiver operating characteristic curve (AUC) of the test set was 0.76.

**Fig. 1:**
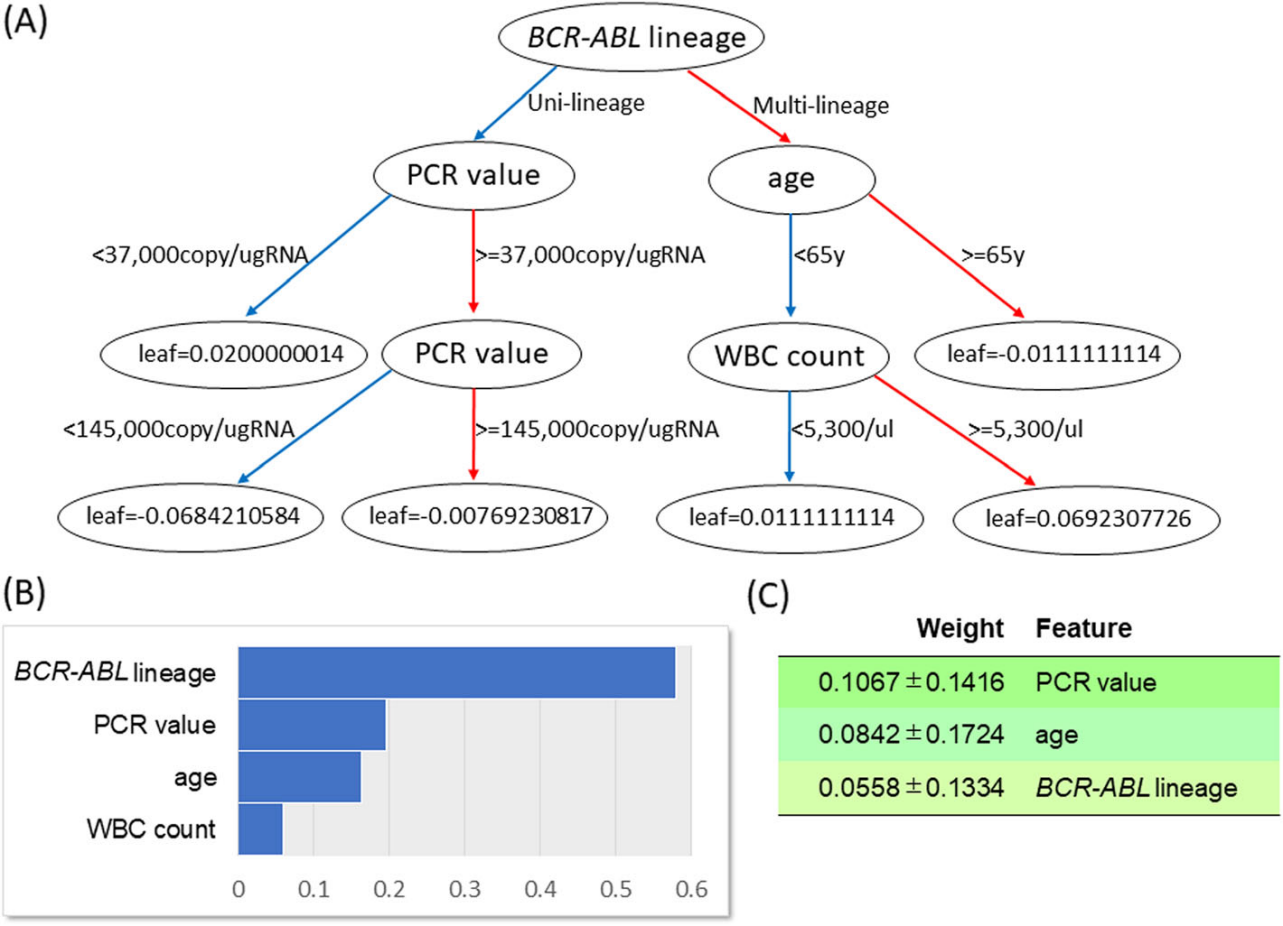
XGBoost model. **a** XGBoost plot of a single decision tree. **b** The feature importance score. **c** The permutation feature importance

In multivariate analysis using the conventional Cox model, *BCR-ABL* lineage and age were identified as significant risk factors [6]. According to the feature importance score, two more factors, polymerase chain reaction (PCR) value and white blood cell (WBC) count, were also identified as important features, and the XGBoost decision tree used these four factors as nodes, which suggested these four features were important for the model construction (Fig. 1a and b). There were no strong correlations between the features: the absolute value of the correlation coefficients was between 0.016 (*BCR-ABL* value and PCR value) and 0.27 (PCR value and WBC count). The mean variance inflation factor for checking multicollinearity between WBC count and another feature was 1.06 (range 1.01–1.09). The permutation feature importance also showed that PCR value, age, and *BCR-ABL* lineage were important features, which was indicative of how much the prediction using the test set depended on these features (Fig. 1c). The AUC, sensitivity, and specificity were 0.77 [Standard error (SE) 0.06], 0.59, and 0.89 when using parameters identified in the XGBoost model, and 0.72 (SE 0.06), 0.50, and 0.81 when using those identified in the conventional COX model. In the XGBoost model for predicting an event within 2 years from diagnosis, *BCR-ABL* lineage, PCR value, age, and WBC count were also identified as important features according to the feature importance score (Fig.S1A). The permutation feature importance also identified these four features as important (Fig.S1B).

## Survival stratification

**Fig. 2:**
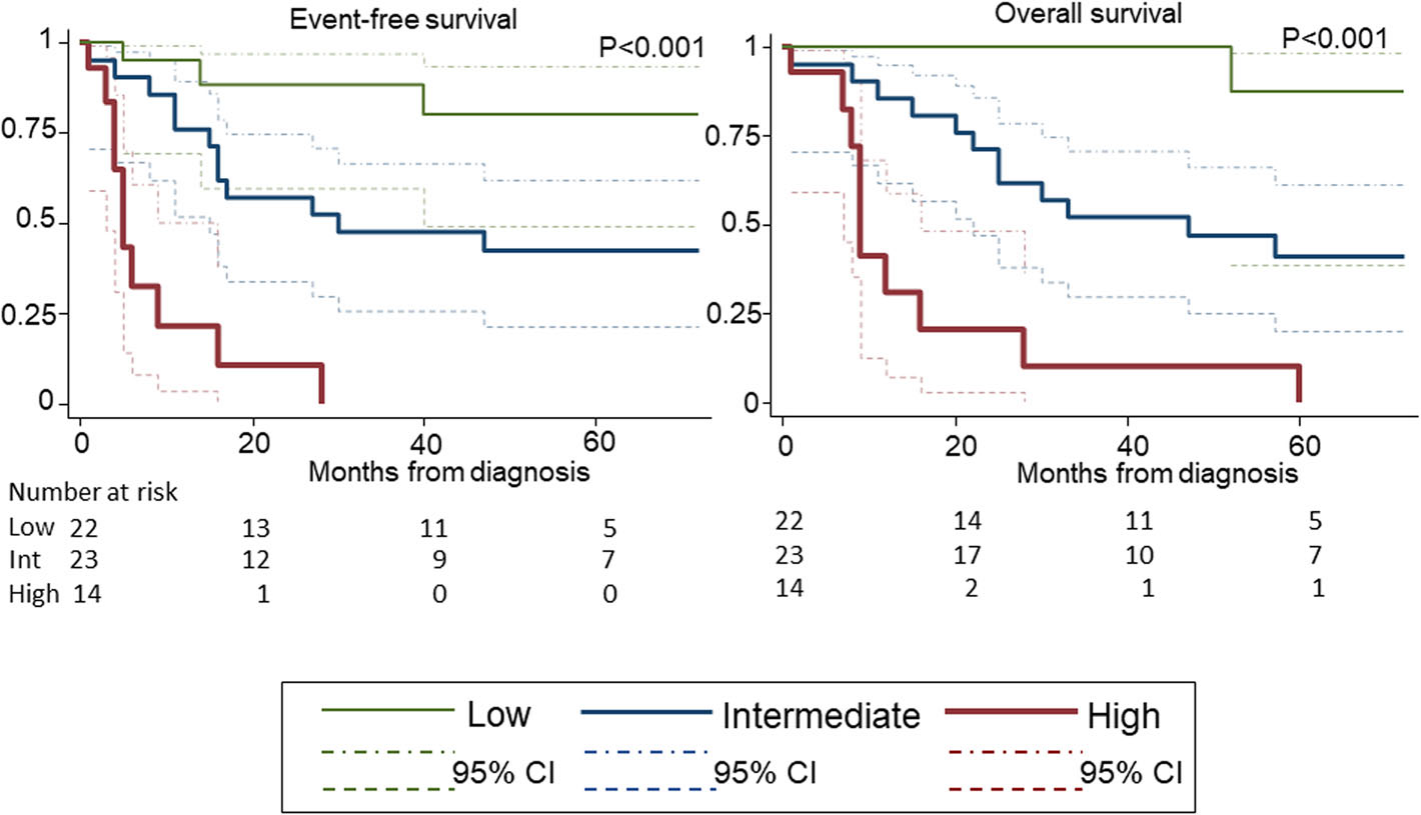
Survival stratification. Survival was stratified by risk groups using four important features (*BCR-ABL* lineage, PCR value, age and WBC count) identified by the XGBoost model. Low, two or less factors; Int, three factors; High, four factors. **a** Event-free survival (EFS). **b** Overall survival (OS). The EFS and OS were 88% (95%CI 60–97%) and 100% in Low, 57% (34–75%) and 71% (47–86%) in Int, and 11% (0.6–38%) and 21% (3–49%) in High at 2 years, and 80% (49–93%) and 100% in Low, 42% (21–62%) and 47% (25–66%) in Int, and 0 and 10% (0.6–37%) in High, respectively at 4 years

Based on the index of dichotomy in the XGBoost decision tree, we considered the following four features as risk factors: uni-lineage Ph leukemia (uni-Ph), a *BCR-ABL* PCR value≥14500copies/μgRNA, age ≥ 65 years, and WBC count ≥5300/μl. The cohort was divided into three risk groups according to the number of risk factors: low-risk group (Low; two or less factors), intermediate-risk group (Int; three factors), and high-risk group (High; four factors) (TableS3). The event-free survival (EFS) and overall survival (OS) were compared among the three risk groups using conventional statistical techniques (TableS4). The EFS and OS were 80 and 100% in Low, 42 and 47% in Int, and 0 and 10% in High, respectively at 4 years (Fig. 2). The same trend was also confirmed in the stratification using only the test set: EFS at 4 years was 100% in Low, 80% (20–97%) in Int, and 0% in High (*P* = 0.046).

## Discussion

The advantage of extracting risk factors using machine learning is that it can reduce the influence of artificial variable selection that can occur in conventional statistical analyses. In addition, new factors that go unnoticed by humans may be extracted. In this study, the PCR value of *BCR-ABL* was identified as an important feature. The PCR value of *BCR-ABL* is considered to be important for following MRD in Ph + ALL [2, 8–11], so it is not common to consider PCR value at diagnosis as a risk factor in conventional analyses. It is interesting that such a new factor was identified as being useful for prognostic stratification.

In this study, the XGBoost algorithm could extracted clinically valid features using a small dataset comprising 59 cases. Since the small number of cases was one of the major limitations of this study, additional confirmation is required to validate the methodology. Although the difference in predictive indices was small between conventional and machine learning-aided methods, it was suggested that the new parameters could contribute to improving each index.

## Supplementary Information

**Additional file 1:** **Fig. S1.** Important feature for event within 2 years. (A) The feature importance score. (B) The permutation feature importance.

**Additional file 2.** Supplementary methods.

## Data Availability

All data generated or analysed during this study are included in this published article.

## Abbreviations

AUC: area under the receiver operating characteristic curve
EFS: event-free survival
High: high-risk group
Int: intermediate-risk group
Low: low-risk group
MRD: minimal residual disease
OS: overall survival
Ph + ALL: Philadelphia chromosome-positive acute lymphoblastic leukemia
PCR: polymerase chain reaction
SE: Standard error
uni-Ph: uni-lineage Ph leukemia
WBC: white blood cell
XGBoost: eXtreme Gradient Boosting
