# Supplementary figures and images for: Machine learning-aided risk stratification in Philadelphia chromosome-positive acute lymphoblastic leukemia
Source: Biomark Res. 2021 Feb 18;9:13. doi: 10.1186/s40364-021-00268-x (PMC7890949; doi:10.1186/s40364-021-00268-x)

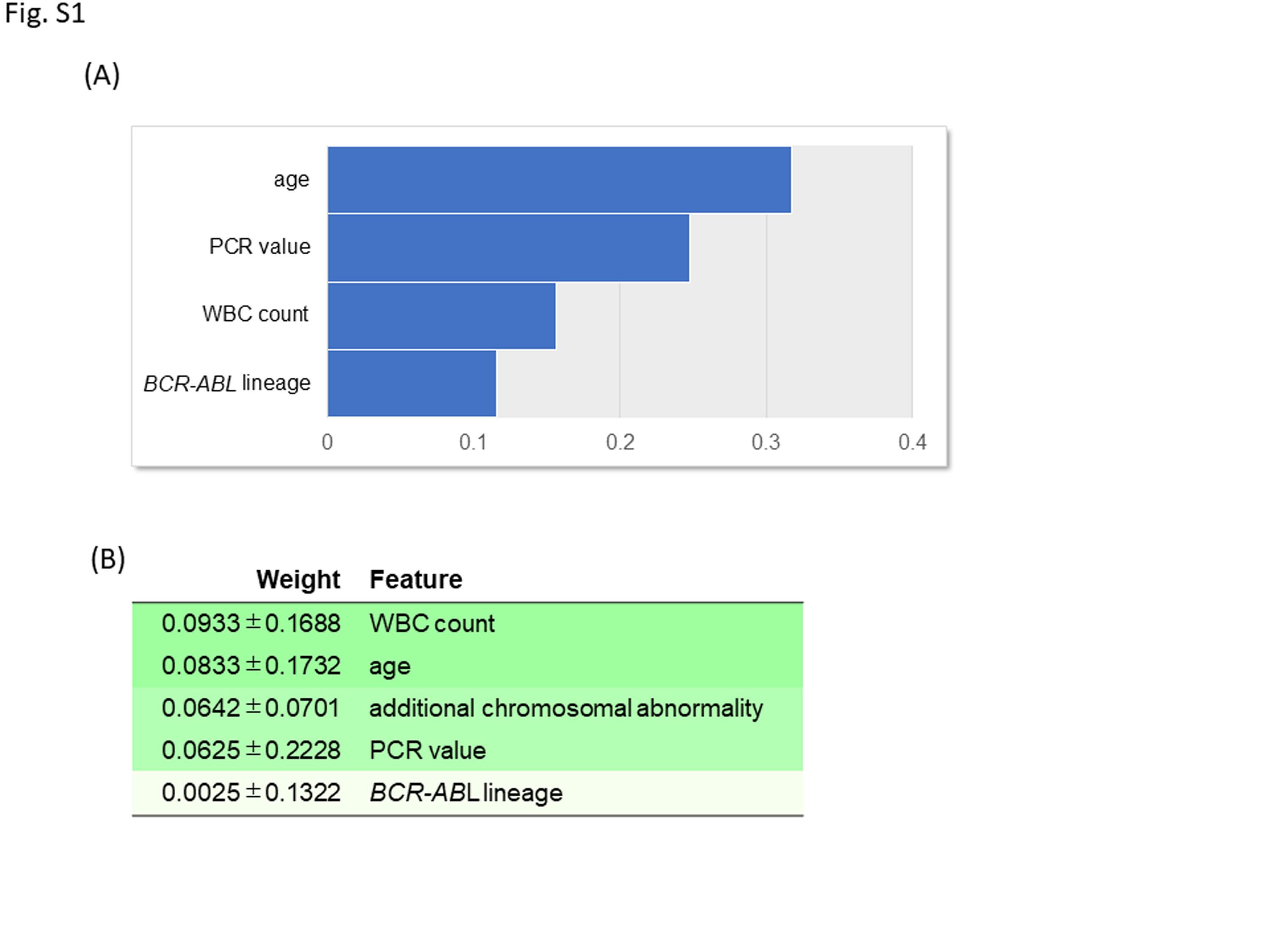

Supplement: Supplementary file 1 — Additional file 1: Fig. S1. Important feature for event within 2 years. (A) The feature importance score. (B) The permutation feature importance. [file 40364_2021_268_MOESM1_ESM.tif]
