# Supplementary material for: Machine learning-aided risk stratification in Philadelphia chromosome-positive acute lymphoblastic leukemia
Source: Biomark Res. 2021 Feb 18;9:13. doi: 10.1186/s40364-021-00268-x (PMC7890949; doi:10.1186/s40364-021-00268-x)
Supplement: Supplementary file 2 — Additional file 2. Supplementary methods. [file 40364_2021_268_MOESM2_ESM.docx]

**Supplementary methods**

**Study population and definition of covariates**

Using a dataset of 59 adult Philadelphia chromosome-positive acute lymphoblastic leukemia (Ph+ALL) patients that we previously used to clarify the importance of the *BCR-ABL* expressing lineage in Ph+ALL [1], we attempted to identify further risk factors by performing machine learning-aided analysis. Through the establishment of a predictive model of whether an event will occur, important features were identified. An event was defined as death by any cause, failure to achieve remission after induction therapy, relapse in any site, or second malignancy [2-4]. Multi-lineage *BCR-ABL* expression (multi-Ph) was defined as *BCR-ABL* expression outside the B-lineage compartment, which was identified by a positive result in peripheral blood neutrophils by fluorescence in situ hybridization (FISH), FISH in bone marrow clot section, and/or flow cytometry-sorted polymerase chain reaction (PCR), as previously described [1]. Otherwise, it was judged as uni-lineage Ph leukemia (uni-Ph). The copy numbers of *BCR-ABL* transcripts in each sample were reported as normalized values by means of *GAPDH*. The threshold for quantification was 50 copies/μg RNA, which corresponded to a minimal sensitivity of 10^-5^ [5]. In the present study, “not detected” by real-time quantitative PCR was defined as complete molecular remission. All patients were treated with tyrosine kinase inhibitor ± conventional chemotherapy. Written informed consent was obtained from each patient upon treatment and sample collection. Each hospital’s institutional review board approved the study.

**Machine learning algorithm**

We used the eXtreme Gradient Boosting (XGBoost) model, which is an implementation of gradient boosted decision trees and an optimized gradient boosting machine learning library [6]. The data used for predictive modeling are listed in Table S1. We randomly separated the data into a training set and a test set (test size=0.2) (TableS2). The test set was only used to assess the performance of the trained model. We tuned the XGBoost hyperparameters using a grid search with cross validation. A learning rate of 0.05 and colsample bytree of 1.0 were selected as tuned parameters. The validation of the tuned model was performed using three-fold cross validation. The feature importance score was calculated based on so-called “gini importance” or “mean decrease impurity”, which was defined as the total decrease in node impurity averaged over all trees of the ensemble. The permutation feature importance, also known as “mean decrease accuracy”, was computed based on the test set, and was defined as the decrease in a model score when a single feature value was randomly shuffled [7]. The cut-off is choosing that value that minimizes the Euclidean distance between the receiver operating characteristic curve and the upper left corner of the graph. To consider the effect of time-to-event, the feature importance score and permutation feature importance for events within two years from diagnosis were also evaluated, as was the evaluation of feature importance for all events. The model was developed in Python version 3.7.6 using the packages scikit-learn version 0.23.0 and py-xgboost version 0.90.

**Survival analysis**

The probabilities of event-free survival (EFS) and overall survival (OS) were estimated from the diagnosis using the Kaplan–Meier method. *P-*values were calculated using a log-rank test [8, 9] and compared using the Cox proportional hazard regression model [10], according to the risk groups determined by the XGBoost algorithm. Stratification was performed based on the important features identified in the XGBoost model. A significance level of *P* <0.05 was used for all analyses. Stata version 13.1 (Stata Corp., College Station, TX) was used for conventional statistical analyses.

**References**

1. Nishiwaki S, Kim J, Ito M, Maeda M, Okuno Y, Koyama D, Ozawa Y, Gunji M, Osaki M, Kitamura K *et al*: Multi-lineage BCR-ABL Expression in Philadelphia Chromosome-positive Acute Lymphoblastic Leukemia is Associated with Improved Prognosis but no Specific Molecular Features. Front Oncol 2020;10:586567.

2. Butturini AM, Dorey FJ, Lange BJ, Henry DW, Gaynon PS, Fu C, Franklin J, Siegel SE, Seibel NL, Rogers PC *et al*: Obesity and outcome in pediatric acute lymphoblastic leukemia. J Clin Oncol 2007;25:2063-2069.

3. Nachman JB, La MK, Hunger SP, Heerema NA, Gaynon PS, Hastings C, Mattano LA, Jr., Sather H, Devidas M, Freyer DR *et al*: Young adults with acute lymphoblastic leukemia have an excellent outcome with chemotherapy alone and benefit from intensive postinduction treatment: a report from the children's oncology group. J Clin Oncol 2009;27:5189-5194.

4. Schultz KR, Carroll A, Heerema NA, Bowman WP, Aledo A, Slayton WB, Sather H, Devidas M, Zheng HW, Davies SM *et al*: Long-term follow-up of imatinib in pediatric Philadelphia chromosome-positive acute lymphoblastic leukemia: Children's Oncology Group study AALL0031. Leukemia 2014;28:1467-1471.

5. Mizuta S, Matsuo K, Nishiwaki S, Imai K, Kanamori H, Ohashi K, Fukuda T, Onishi Y, Miyamura K, Takahashi S *et al*: Pretransplant administration of imatinib for allo-HSCT in patients with BCR-ABL-positive acute lymphoblastic leukemia. Blood 2014;123:2325-2332.

6. Chen T, Guestrin C: XGBoost: A Scalable Tree Boosting System. arXiv:160302754v3 [csLG] 10 Jun 2016 2016.

7. Wang H, Yang F, Luo Z: An experimental study of the intrinsic stability of random forest variable importance measures. BMC Bioinformatics 2016;17:60.

8. Kaplan EL, Meier P: Nonparametric estimation from incomplete observations. J Am Stat Assoc 1958;53:457-481.

9. Peto R, Peto J: Asymptotically efficient rank invariant test procedures. J R Stat Soc A 1972;135:185-207.

10. Cox D: Regression models and life tables. J R Stat Soc B 1972;34:187-220.

**Table S1** Variables used for the XGBoost model

| Sex |
| --- |
| Age |
| WBC count at diagnosis |
| Bone marrow blasts percentage at diagnosis |
| PCR value of *BCR-ABL* at diagnosis |
| *BCR-ABL* expression lineages (uni-Ph / multi-Ph) |
| *BCR-ABL* transcript (e1a2 / b2a2 or b3a2) |
| Additional cytogenetic abnormality (Yes / No / unknown) |
| Tyrosine kinase inhibitor(Imatinib / Dasatinib) |
| Complete molecular remission after the first induction (Yes / No) |
| Allogeneic hematopoietic cell transplantation (Yes / No) |

**Table S2** Patient characteristics

|  | Training set | (%) | Test set | (%) | *P* |
| --- | --- | --- | --- | --- | --- |
| No. of patients | 47 |  | 12 |  |  |
| Sex |  |  |  |  | 0.14 |
| Male | 23 | 49 | 3 | 25 |  |
| Female | 24 | 51 | 9 | 75 |  |
| Age | 56 (15-88) |  | 64 (41-92) |  | 0.20 |
| < 65 y | 30 | 64 | 6 | 50 | 0.38 |
| ≥ 65 y | 17 | 36 | 6 | 50 |  |
| WBC at diagnosis /ul [median(range)] | 23080 (1240-730000) | | 24295 (3700-412500) | | 0.84 |
| < 30000 /ul | 25 | 53 | 7 | 58 | 0.75 |
| ≥ 30000 /ul | 22 | 47 | 5 | 42 |  |
| Bone marrow blasts at diagnosis % [median(range)] | 92.5 (20.3-100) | | 94.7 (35.9-98.3) | | 0.47 |
| PCR value of *BCR-ABL* at diagnosis copy/ugRNA [median(range)] | 680000 (0-4800000) | | 510000 (40000-1503409) | | 0.61 |
| *BCR-ABL* transcript |  |  |  |  | 0.62 |
| e1a2 | 31 | 66 | 7 | 58 |  |
| b2a2 or b3a2 | 16 | 34 | 5 | 42 |  |
| Additional cytogenetic abnormality |  |  |  |  | 0.90 |
| (-) | 17 | 36 | 4 | 33 |  |
| (+) | 28 | 60 | 6 | 50 |  |
| Unknown | 2 | 4 | 2 | 17 |  |
| Tyrosine kinase inhibitor |  |  |  |  | 0.39 |
| Imatinib | 28 | 60 | 6 | 50 |  |
| Dasatinib | 16 | 34 | 6 | 50 |  |
| Other | 3 | 6 | 0 | 0 |  |
| Allogeneic hematopoietic cell transplantation | |  |  |  | 0.79 |
| (-) | 25 | 53 | 8 | 67 |  |
| (+) | 22 | 47 | 4 | 33 |  |

**Table S3** Patient characteristics according to the risk group

| Risk | Low | (%) | Intermediate | (%) | High | (%) | *P* |
| --- | --- | --- | --- | --- | --- | --- | --- |
| No. of patients | 22 |  | 23 |  | 14 |  |  |
| Sex |  |  |  |  |  |  | 0.56 |
| Male | 8 | 36 | 12 | 52 | 6 | 43 |  |
| Female | 14 | 64 | 11 | 48 | 8 | 57 |  |
| Age | 52 (15-67) |  | 61 (34-79) |  | 66 (47-92) |  | <0.001 |
| < 65 y | 22 | 100 | 14 | 61 | 0 | 0 | <0.001 |
| >= 65 y | 0 | 0 | 9 | 39 | 14 | 100 |  |
| WBC at diagnosis /ul [median(range)] | 20530  (1240-730000) | | 16300  (1900-412500) | | 56190  (5650-199600) | | 0.59 |
| < 30000 /ul | 13 | 59 | 14 | 61 | 5 | 36 | 0.28 |
| >= 30000 /ul | 9 | 41 | 9 | 39 | 9 | 64 |  |
| Bone marrow blasts at diagnosis % [median(range)] | 86.1 (20.3-99.5) | | 94.4 (66.4-100) | | 92.4 (62-98.7) | | 0.08 |
| PCR value of BCR-ABL at diagnosis copy/ugRNA [median(range)] | 490000  (0-2600000) | | 575000 (160000-2300000) | | 1200000  (185000-4800000) | | 0.11 |
| *BCR-ABL* transcript |  |  |  |  |  |  | 0.18 |
| e1a2 | 11 | 50 | 16 | 70 | 11 | 79 |  |
| b2a2 or b3a2 | 11 | 50 | 7 | 30 | 3 | 21 |  |
| Additional cytogenetic abnormality |  |  |  |  |  |  | 0.15 |
| (-) | 11 | 50 | 5 | 22 | 5 | 36 |  |
| (+) | 11 | 50 | 17 | 74 | 6 | 43 |  |
| Unknown | 0 | 0 | 1 | 4 | 3 | 21 |  |
| Tyrosine kinase inhibitor |  |  |  |  |  |  | 0.07 |
| Imatinib | 10 | 45 | 13 | 57 | 11 | 79 |  |
| Dasatinib | 12 | 55 | 8 | 35 | 2 | 14 |  |
| Other | 0 | 0 | 2 | 9 | 1 | 7 |  |
| Allogeneic hematopoietic cell transplantation | |  |  |  |  |  | 0.13 |
| (-) | 10 | 45 | 12 | 52 | 11 | 79 |  |
| (+) | 12 | 55 | 11 | 48 | 3 | 21 |  |

**Table S4** Validation analysis of risk groups by XGBoost model

| Risk group | Event-free survival | | |  | Overall survival | | |
| --- | --- | --- | --- | --- | --- | --- | --- |
|  | HR | 95%CI | *P* |  | HR | 95%CI | *P* |
| Low |  | Reference |  |  |  | Reference |  |
| Intermediate | 3.84 | 1.08-13.6 | 0.04 |  | 10.3 | 1.34-79.3 | 0.03 |
| High | 18.9 | 4.92-72.9 | <0.001 |  | 41.4 | 5.21-329.3 | <0.001 |
